# Quantum Tunnelling to the Origin and Evolution of Life

**DOI:** 10.2174/13852728113179990083

**Published:** 2013-08

**Authors:** Frank Trixler

**Affiliations:** 1 Center for NanoScience (CeNS), Ludwig-Maximilians-Universität München, Schellingstraße 4, 80799 München, Germany;; 2 Department of Earth and Environmental Sciences, Ludwig-Maximilians-Universität München, Theresienstrasse 41, 80333, München, Germany;; 3 TUM School of Education, Technische Universität München, Museumsinsel 1, 80538 München, Germany

**Keywords:** Biomolecular nanomachines, Interstellar, Life, Nucleosynthesis, Planetary habitability, Prebiotic, quantum tunnelling, Radioactive decay.

## Abstract

Quantum tunnelling is a phenomenon which becomes relevant at the nanoscale and below. It is a paradox from the classical point of view as it enables elementary particles and atoms to permeate an energetic barrier without the need for sufficient energy to overcome it. Tunnelling might seem to be an exotic process only important for special physical effects and applications such as the Tunnel Diode, Scanning Tunnelling Microscopy (electron tunnelling) or Near-field Optical Microscopy operating in photon tunnelling mode. However, this review demonstrates that tunnelling can do far more, being of vital importance for life: physical and chemical processes which are crucial in theories about the origin and evolution of life can be traced directly back to the effects of quantum tunnelling. These processes include the chemical evolution in stellar interiors and within the cold interstellar medium, prebiotic chemistry in the atmosphere and subsurface of planetary bodies, planetary habitability via insolation and geothermal heat as well as the function of biomolecular nanomachines. This review shows that quantum tunnelling has many highly important implications to the field of molecular and biological evolution, prebiotic chemistry and astrobiology.

## 1 INTRODUCTION

Quantum mechanical phenomena are not comprehensible via concepts and assumptions derived from the macroscopic world. For example, the daily experience that things have a definitive property, are located at a certain time on a definite place and have a definite energy doesn't help when we zoom in to the scale of nanometres and below. At this scale, when trying to understand why reactions between elementary particles, nucleons or atoms occur at energies which would classically not allow these reactions to occur, we arrive at a paradox. Quantum mechanics, however, resolves this paradox. The way it does this guides us to a fascinating phenomenon: tunnelling. This review sheds light on the importance of the tunnelling phenomenon for many processes essential in theories of the origin and evolution of life such as prebiotic chemistry, chemical evolution, biochemical reactions and the formation of habitable planetary environments.

**Fig. (1):**
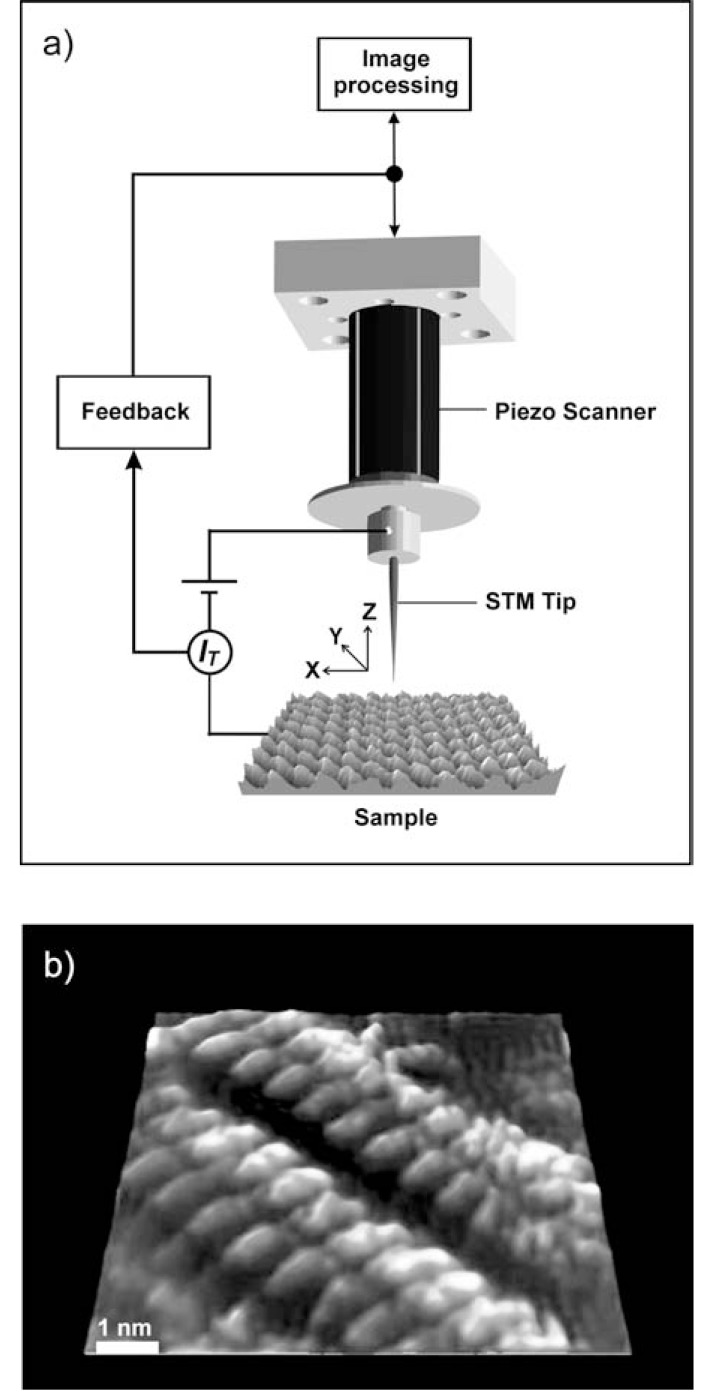
Scanning Tunnelling Microscopy (STM). a) Operation principle. The sample is a 3D visualization of a graphite surface imaged via STM with atomic resolution. b) STM image of supramolecular chains of organic molecules adsorbed on a mineral surface (graphite). The linear chains are formed through the interaction of Acridone molecules via hydrogen bonds. Acridone is structurally analogue to Anthracene, a polycyclic aromatic hydrocarbon which has been found in molecular clouds of the interstellar medium (see section 3). Life uses structural analogues of Anthracene and Acridone as functional subunits in complex biomolecules (e.g. Flavin, see section 5).

Now, what is quantum tunnelling? A brief introduction into this strange phenomenon becomes more illustrative when referring to one of its technological applications. A good example is Scanning Tunnelling Microscopy (STM) [1-3] as it illustrates both the preconditions and the nature of quantum tunnelling. STM enables us to directly obtain images of surfaces with atomic or sub- molecular resolution with comparatively low instrumental effort (Fig. **1**). In order to obtain such images, a sharp metallic tip is placed within a distance of about one nanometre or less above an electrically conductive sample surface and a voltage is applied between the tip and the surface. The tip is moved above the surface by a piezoelectric actuator in a raster scanning pattern in order to map tiny variations of the resulting electrical current. These variations are caused by the local density of electronic states in the substrate surface and/or by the topography of the surface. Atomic resolution of crystalline surfaces is easily possible due to the extreme sensitivity of the measured current on the tip-sample distance. This sensitivity is so high that it causes a detectable difference in current when moving the tip from a position above the valley between adjacent atoms to a position exactly above an atom. The trick to achieving such an enormous sensitivity is the phenomenon of quantum tunnelling.

The central characteristic of quantum tunnelling is the fact that under certain conditions elementary particles, nucleons or atoms are able to negotiate the obstacle of a potential barrier (which is, from the classical point of view, a forbidden area for a particle) without having the energy to overcome it. The STM setup gives an illustrative example of potential barrier width: the nanometre-wide gap between the tip and the sample. The benefit of creating such a gap becomes clear when considering the nature of quantum tunnelling.

According to the standard interpretation of quantum mechanics, the quantum state of material particles such as electrons can be described by a wave function Ψ. It seems strange to describe the state of a material particle by a wave function. However, this refers to one of the central postulates of quantum mechanics: wave-particle duality. It has been postulated that particles also have a wave character based on the observation that the detected character of a material particle (corpuscle or wave) depends solely on the experimental design. Therefore a particle can be described as a matter-wave [4]. A matter-wave can mathematically be represented by a wave function which is the solution of a wave equation. The appropriate wave equation for material particles is the Schrödinger equation [5-8] whose solution describes the behaviour of a particle in space and time (e.g. with respect to a potential barrier). It is therefore this equation which helps to explain the quantum tunnelling phenomenon.

**Fig. (2):**
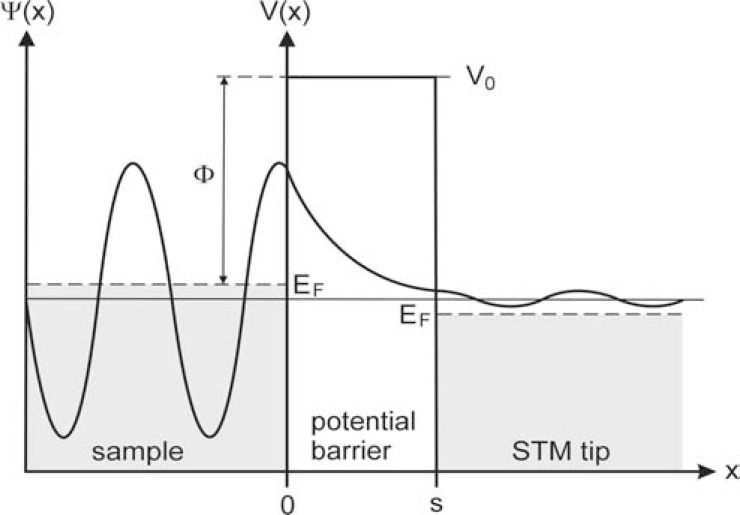
Quantum tunnelling effect. Illustrated is the exponential damping of a wave function within a one-dimensional potential barrier (after [3], modified), with Φ as the tunnelling barrier height (in the case of STM this barrier height is the effective local work function: the energy required to remove an electron from the sample electrode to bring it to the vacuum level V_0_), E_F_ as the Fermi levels of the metal electrodes and s as the barrier width (extending from x=0 to x=s).

In the case of an electron faced with a gap between a STM tip and a sample we can use a simple model of a one-dimensional rectangular potential energy barrier with limited height. This model is sufficient to explain the nature of quantum tunnelling in a qualitative way. Based on this model we can write the time-independent, one-dimensional Schrödinger equation [9,10] as

(1) $$-\frac{\hbar^{2}}{2m}\frac{\delta^{2}\Psi \left(x\right)}{\delta x^{2}}+V\left(x\right)\Psi \left(x\right)=E\Psi \left(x\right)$$

where *m* is the particle mass (in our case the mass of an electron), *ћ* is the reduced Planck constant, *V* is the potential energy and *E *is the total energy of a particle. The differential operator in this equation is connected with the kinetic energy. Within the rectangular potential barrier of height *V_0_* extending from *x*=0 to *x*=*s* (Fig. **2**) the solution of this equation reads

(2) $$\Psi \left(x\right)=\Psi \left(0\right)e^{-\mathrm{kx}}$$

for a high (*V_0_* > *E*) and wide barrier (*ks* > 1). Here *s* denotes the barrier width (the tip-sample distance in STM) and *k* denotes the decay constant of a particle state in the potential barrier region (in our example: an electron state near the Fermi level E_F_ of the metallic sample), where

(3) $$k=\frac{\sqrt{2m\Phi }}{\hbar }$$

with *Φ *as the local tunnelling barrier height (*Φ=V_0_-E_F_*). In the case of STM this tunnelling barrier height is the effective local work function or, in other words, the minimum energy required to remove an electron from the metal electrode (e.g. the STM sample) and to bring it to the vacuum level [1,3].

Equation (2) tells us that within a potential barrier there is an exponential damping of the wave function along the positive *x* direction. (Fig. **2**) illustrates this damping for the example of a STM situation. The fact that the wave function within the potential barrier (*0 ≤ x ≤ s*) is just damped and not erased altogether has an important consequence: as long as the barrier width is not too large there is still a small amplitude of the wave function behind the barrier. What does that mean? Although the wave function Ψ reflects the wave nature of a particle, it is complex-valued and therefore doesn't provide a measurement parameter. However, the square of the absolute value of the wave function (|Ψ_(x)_|^2^) can statistically be interpreted as the probability density for a particle to be detected at a location point *x* [e.g. 10]. Therefore a remaining amplitude of the wave function behind the barrier means that there is a small but nonetheless existing probability for a particle to ''transmit'' or ''tunnel'' through the barrier even if there is no sufficient energy to overcome the barrier classically. The transmission probability (*P*) can be calculated via the equation

(4) $$P=\frac{{\left|\Psi_{\left(s\right)}\right|}^{2}}{{\left|\Psi_{\left(0\right)}\right|}^{2}}=e^{-2\mathrm{ks}}$$

and provides a clear measurement parameter. In fact a STM measures a current which is proportional to the exponential *e*^-2ks^. The value of the current depends not only on the local electron density of states in the sample and the tip but also corresponds to the transmission probability of an electron passing the tip-sample barrier. It is this exponential dependence of the transmission probability on the width and height of a potential barrier which causes the extreme sensitivity of the resulting current to the tip-sample distance. The exponential dependence enables gaining images with atomic resolution because tiny changes in the tip-sample distance cause large changes in the resulting tunnelling current. This is why a nanometre-wide tip-sample gap in STM is so important: it creates a situation characteristic of quantum tunnelling by measuring the result of quantum mechanical transmission probabilities.

The Schrödinger equation not only enables a qualitative description of quantum tunnelling, but is also essential for more advanced, quantitative models of tunnelling (for a most recent overview of such tunnelling models for STM see [11]). However, it is not the only possible way to explain and describe the tunnelling phenomenon. Let us remember what the Schrödinger equation is based on: the wave-particle dualism. As this dualism is among the central postulates of quantum mechanics there are other important principles which are also related to it. One of these principles is the Uncertainty Principle (Heisenberg principle) [12,13]. A relation between the Uncertainty Principle and the wave-particle dualism arises by the fact that this principle applies to all wave-like systems, including matter waves. 

The Uncertainty Principle refers to complementary properties of a particle such as position/momentum or energy/time and states that the values of such conjugated variables cannot be known simultaneously to arbitrary accuracy. What are the consequences of this fundamental limit of precision and how can this principle explain that particles are able to negotiate a potential barrier without having the energy to overcome it? Let us focus on the example of energy-time uncertainty. It can be written as

(5) $$\Delta E\Delta t\approx \frac{\hbar }{2}$$

with Δ*E* as the uncertainty in energy and Δ*t* as the uncertainty in time. As the right part of (5) is a constant, the relation implies that e.g. the shorter the time Δ*t *is, the more the energy of a particle becomes uncertain (Δ*E*) and vice versa. Based on this relation the tunnelling phenomenon can be explained as follows: if the time Δ*t* is sufficiently short, the energy of a particle *E* + Δ*E* can exceed the height of the potential barrier and thus the particle can overcome it. In other words, quantum tunnelling occurs if a particle is able to negotiate a potential barrier within the time Δ*t. *With respect to the model of a rectangular potential barrier of height *V_0_* and width *s* this time can be estimated via the following relation [3,14]:

(6) $$\Delta t=\frac{s}{\sqrt{\left(\frac{2}{m}\right)\left(E+\Delta E-V_{0}\right)}}$$

Another possible point of view on quantum tunnelling is the position/momentum uncertainty:

(7) $$\Delta x\Delta p\approx \frac{\hbar }{2}$$

It states that the more accurate the momentum of a particle is known, the more the position becomes uncertain and vice versa. As a consequence, tunnelling occurs if the momentum uncertainty Δ*p *and thus the uncertainty in kinetic energy of a particle is so large that it can overcome the energy barrier [14]. We see that quantum tunnelling can be explained in multiple ways based on the wave-particle dualism.

The central messages about the tunnelling phenomenon can be summarized as follows: Quantum tunnelling enables particles to manage a potential barrier when there is insufficient energy to overcome it. This phenomenon can be explained by referring to wave-particle duality and can be described on an exact mathematical basis. It is of probabilistic nature and shows an exponential probability decay with increasing barrier width.

Quantum tunnelling is not such a rare phenomenon as it might seem when looking at its preconditions and characteristics. The following sections will demonstrate that it is of vital importance for life in many aspects.

## 2 THE IMPORTANCE OF QUANTUM TUNNELLING FOR LIFE IN INSOLATION HABITABLE ZONES

Chemical and biological evolution requires a steady stream of energy. Free energy is necessary to generate self-organized, adaptable molecular and living systems and to keep them away from thermodynamic equilibrium [15]. The most important source of free energy for life on earth is sunlight as it is the key factor in oxygenic photosynthesis. This type of photosynthesis fuels most ecosystems directly (phototrophy) as well as indirectly (chemotrophy based on energy from organic matter produced by oxygenic photosynthesis). It also enriches the atmosphere and hydrosphere with its byproduct O_2_. The enrichment with O_2 _forced the development of aerobic cellular respiration, which was the major precondition for the evolution of complex, metazoan life (multicellular animals). This is because the complexity, size and mobility of metazoans requires a very high amount of free energy compared to other lifeforms, and the reduction of O_2_ by aerobic cellular respiration releases about an order of magnitude more energy per intake of food than other types of energy metabolism [16]. Therefore, it is the input of solar photon energy (insolation) used by oxygenic photosynthesis which energetically enabled the evolution of complex, multicelluar life on earth.

A long period of time is also required for chemical and subsequent biological evolution of complex life. It took billions of years to achieve major steps in the origin and evolution of life on earth. According to the geological and paleontological record and recent age determinations, the complete accretion and major early differentiation of the earth occurred around 4.5 – 4.4 billion years (Ga) ago [17]. The oldest evidences for bacterial life date back to 3.4 Ga [18,19], the oldest unambiguous eucaryotic microfossils to 1.8 Ga [20], the oldest fossils of metazoa to 0.63 Ga [21] and the oldest known fossils of anatomically modern Homo *sapiens* to 0.00016 Ga [22]. Thus, a constant solar energy flux over billions of years was a precondition for the biological evolution towards complex life on earth. But what has this all to do with quantum tunnelling?

Solar energy production is based on the fusion of lighter atomic nuclei to heavier ones [23]. A fusion reaction releases nuclear binding energy. The released binding energy is equivalent to a mass deficit according to Einsteins energy-mass equivalence *E=mc^2^* [24] and therefore the mass of the fusion product (the heavier nucleus) is smaller than the sum of the individual masses of the lighter nuclei. But what enables a nuclear fusion reaction within a star? As each nucleus of the stellar plasma has a positive nuclear charge, there is a high repulsive electrostatic force between the nuclei. Thus, for a nuclear fusion, a high amount of energy is required to bring atomic nuclei close enough to each other (distance *r_c_* ≤ 10^-15 ^m) to overcome the repulsive Coulomb force so that they are tied up by the strong interaction. The only energy available in a star to potentially overcome the Coulomb barrier is the kinetic energy of the nuclei due to thermal motion [25]. The central temperature of stars is within the regime of 10^7 ^K [26] – a value for which the assumption of an ideal gas is justified [23]. Therefore, the average kinetic energy of a nucleus can be calculated via

(8) $$E_{\mathrm{kin}}=\frac{3}{2}\mathrm{kT}$$

where *k* is the Boltzmann constant and *T* is the temperature. For a temperature of 10^7 ^K within the center of of a star *E_kin_* is within the regime of 1 keV. But is this average kinetic energy sufficient to exceed the Coulomb barrier height? The energy *E_cb_* which is needed to overcome the Coulomb barrier height is

(9) $$E_{\mathrm{cb}}=\frac{Z_{1}Z_{2}e^{2}}{r_{c}}$$

where *Z_i_e* is the positive nuclear charge of each nucleus *i *[23,25]. Since the values of *E_cb_* are in the regime of MeV [27], the average kinetic energies available by the solar core temperature are more than a factor of 1000 *below* the necessary energy to overcome the Coulomb barrier. This difference is so high that even though there are nuclei with a larger than average kinetic energy (according to the Maxwell–Boltzmann distribution) the probability of having even one nucleus in the whole solar plasma with a kinetic energy 1000 times above average is virtually zero [25]. As there is no way for atomic nuclei in the stellar interior during quiescent burning to overcome the Coulomb barrier, there must be something additional to thermal motion to make a thermonuclear reaction probable.

This is where quantum tunnelling comes in. Although thermal motion is by far too low in stellar interiors during quiescent burning to directly induce thermonuclear reactions, it is sufficiently high to bring colliding nuclei so close to each other that a significant probability density behind the Coulomb barrier arises: quantum tunnelling occurs with some probability and enables nuclei to penetrate the Coulomb barrier far below the necessary kinetic energy needed to overcome it [28]. Thus, tunnelling is essential for thermonuclear reactions which would otherwise not occur in stars [23,29,30]. This implies that insolation habitable worlds exist due to quantum tunnelling because stellar energy production during quiescent burning can only occur via this phenomenon.

But there is an additional story. We have seen that the evolution of complex eucaryotic, multicellular life on earth required a steady insolation over billions of years. What makes such a long duration of sunlight production possible? Let´s have a closer look at the tunnelling probability *P_t_.* It is proportional to

(10) $$P_{t}\left(V\right)\propto \exp\left(\frac{-4\pi^{2}Z_{1}Z_{2}e^{2}}{h}\frac{1}{v}\right)$$

with *v* as the velocity of the nuclei. This formula [25] shows that the tunnelling probability decreases steeply with lower thermal velocities and with a higher product of the charges *Z_i_e^2^*. One important consequence is that at relatively low stellar core temperatures (as in the case of the sun) a significant tunnelling probability arises only for nuclei pairs with a low value of *Z_i_e^2^*. The lowest value of *Z_i_e^2^* is given for reactions between hydrogen nuclei (protons). For proton-proton reactions (pp-reaction) at solar core conditions *P_t_* is of the order of 10^-20^ [23]. Such a low value of the tunnelling probability gives an indication of the long lifetime which the sun and lower mass stars have on the “main sequence” (quiescent hydrogen burning within the stellar core). However, the tunnelling probability is not equal to the reaction probability. Other factors which are of nuclear origin are also relevant for a reaction to occur, such as the conversion of protons into neutrons via the β^+^-decay.

The reaction probability is mainly determined by the nuclear reaction cross section and the velocity distribution of the atomic nuclei [29]. The reaction cross section *σ(E)* for nonresonant reactions is given by

(11) $$\sigma \left(E\right)=S\left(E\right)E^{-1}\exp\left(-2G\right)$$

with *G* as the Gamow factor, *S(E)* as the Astrophysical S-Factor and *E *as the energy [23]. *S(E) *is of nuclear origin, and the relevant fact for this discussion is that *S(E)* depends only very weak on energy. In contrast, *exp(-2G) * represents the strongly energy dependent part of the cross section because this part is proportional to the tunnelling probability [29].

The astrophysical S-factor *S(E)* influences the value of reaction rates, but is it the tunnelling probability which causes the extreme temperature dependence of the reaction probability. This has an important consequence for low-mass stars, which are the most frequent stars in our galaxy [31,32]: with masses below 1.5 times of the solar mass these stars mainly burn via pp-chain reactions. This is due to the relatively low pressure and thus relatively “low” temperatures within the core. As a consequence of their “low” temperatures, low-mass stars have much smaller reaction cross sections compared to more massive stars because of the strongly energy dependent tunnelling probability. Calculations reveal that at solar core temperature and pressure the reaction cross section of the pp-chain is so small that the mean reaction time for a given proton to react with another proton is 1.4 x 10^10^ years [25]. However, the number of nuclei within the solar interior is so high that there is a sufficient amount of nuclear fusion reactions between protons at any given time to enable a continuous thermonuclear burning. The hydrogen burning continues until all protons in the solar core have been reacted. This will be the case after 1.4 x 10^10^ years of quiescent hydrogen burning - the lifetime of the sun on the main sequence.

In summary, the analysis of the nuclear reaction cross section and the reaction rate reveals that the strong energy dependence of the tunnelling probability causes very small reaction rates within the core of low-mass stars and thus enables a quiescent hydrogen burning over billions of years – a time span which has proven to be necessary for the evolution of complex life on earth.

The importance of quantum tunnelling for life on planets in insolation habitable zones can thus be summarized as follows:

- without quantum tunnelling, thermonuclear reactions would not occur and insolation habitable worlds would not exist
- the low tunnelling probability at the conditions of low-mass stars (such as the sun) “generates” the long duration of sunlight required for the evolution of complex multicellular life on earth and probably on other insolation habitable worlds.

## 3 QUANTUM TUNNELLING AT THE BASIS OF CHEMICAL EVOLUTION

The set of chemical elements which life on Earth uses to build its molecules and to run its biochemistry is relatively small compared to the sum of known elements listed in the periodic table [33, 34]. Besides hydrogen, this small set of elements includes carbon, nitrogen, oxygen, phosphorous, sulphur, sodium, potassium, magnesium and iron. These quantitatively most important elements for life are also among the most abundant heavier elements in the universe and the most common heavier elements in the solar system and sea [35,36]. Their high abundance points to a process of element production which is very prevalent in the universe. This process is nucleosynthesis via thermonuclear reactions in stars [25,28]. As we have seen in section 2 that quantum tunnelling is essential for thermonuclear reactions in stars, the origin of the elements heavier than hydrogen that are most important for life is closely linked to the tunnelling phenomenon.

Despite the hot environment of stellar interiors, the nucleosynthesis process requires quantum tunnelling in order to occur because of insufficient thermal energy for overbarrier fusion reactions. But what is the case when there is a general lack in thermal energy such as in the cold regions of the interstellar medium? What kind of reactions and chemical evolution can occur in these regions – the birthplaces of stars and planetary systems – if the chance of overbarrier reactions is low? Let us shed light on dark clouds and other forms of cold interstellar matter.

**Fig. (3):**
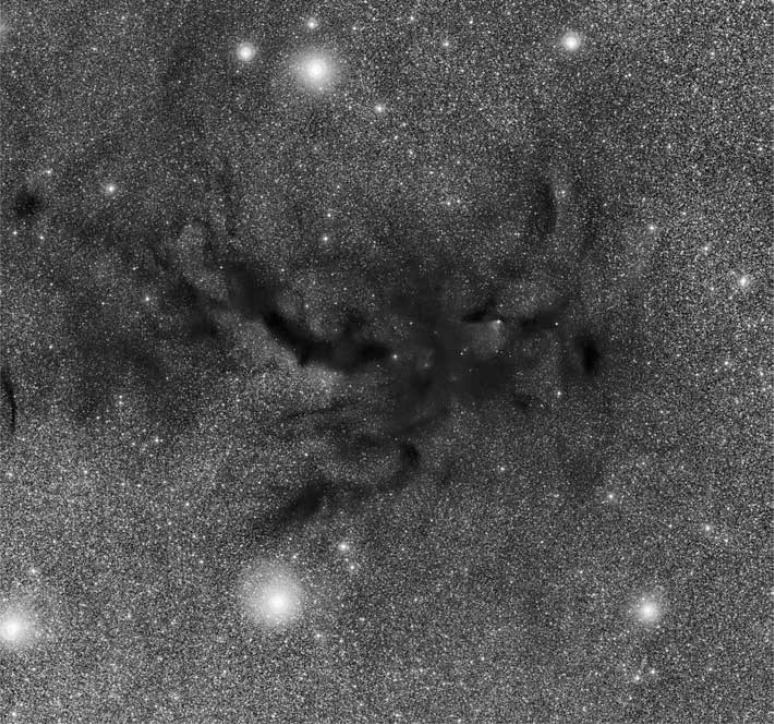
This picture shows Barnard 59, part of a vast dark cloud of interstellar dust called the Pipe Nebula. The image was captured by the Wide Field Imager on the MPG/ESO 2.2-metre telescope at ESO’s La Silla Observatory. Credit: ESO.

 Interstellar matter is the matter between star systems and is composed mainly of hydrogen and helium. The most abundant elements heavier than helium such as carbon, nitrogen, oxygen, magnesium and iron are injected into the interstellar medium mainly by stellar outbursts, stellar winds and by red super-giant stars. These elements account for about 0.1% of the interstellar matter [37]. Dark clouds and neutral diffuse clouds represent the cold part of the interstellar matter with temperatures between 10-100 K and contain micrometer-sized dust grains mixed with the gas [37,38]. The dust content and the density especially of dark clouds is so high (~10^6^ atoms cm^-3^) that electromagnetic radiation is unable to reach the inner region of dense clouds (Fig. **3**) and thus the temperature can drop below 30 K [39].

A rich chemistry takes place in such cold interstellar environments producing a large variety of molecules [40]. Among these molecules H_2_ (molecular hydrogen) is by far the most abundant one [41]. This molecule is very important for various astrochemical and astrophysical processes such as the synthesis of more complex interstellar molecules or the beginning of star formation [42,43]. However, the generation of H_2_ within the interstellar medium has been a long standing problem: the high abundance of H_2 _in neutral diffuse and dark clouds is in contrast to the inefficient gas-phase synthesis routes and destruction by ultraviolet photons and cosmic rays [43,44].

What is enhancing the formation of H_2_ in the interstellar clouds so efficiently? Laboratory experiments and astrochemical models point to the importance of the dust grains mixed with interstellar gas for an efficient interstellar H_2_ synthesis. The importance of dust grains is due to their solid surface which is considered to catalyse the formation of H_2_ [42,45,46]. The first step in this catalytic process is the landing of a hydrogen atom from the gas phase to the morphologically complex surface of a dust grain (adsorption). According to the most familiar route of surface catalysed molecule synthesis, an adsorbed hydrogen atom exchanges energy with the surface and diffuses over the surface until it combines with an already adsorbed hydrogen atom, forming a molecule, which then returns to the gas phase [43].

In dark clouds where the temperature can drop below 20 K the mobility of adsorbed hydrogen atoms is low. On the other hand, the high efficiency of molecular hydrogen production observed in dark clouds indicates a relatively high mobility for adsorbed atoms to come close to each other on the surface via diffusion and to react. What helps to solve this dilemma is quantum tunnelling: thermally assisted tunnelling adds to thermal hopping as a route to enable the required mobility of hydrogen atoms. That means that hydrogen atoms diffuse via tunnelling on the dust grain surfaces in order to find a reaction partner after they have been transported by thermal activation in energy sites where quantum tunnelling is efficient [37,43,45]. This process requires weak interaction forces between the surface and the hydrogen atoms (physisorption) to allow a significant diffusion.

The weak physisorption forces lead to a decrease of efficiency of the described process at higher temperatures as they cannot prevent a fast desorption of H atoms from the surface [42]. However, it has been observed that H_2_ production is also very efficient in neutral diffuse clouds and photon-dominated regions where temperatures are higher (30-200 K). How can this be explained? Observations indicate a quantitative correlation between H_2_ and polycyclic aromatic hydrocarbons (PAHs) especially in photon-dominated regions [47]. According to recent astrochemical models, H_2_ formation in such an interstellar environment can be catalysed by PAHs via chemisorbed H atoms. A chemisorption of H atoms on PAHs means that there are strong interaction forces between the adsorbed H atom and the substrate which significantly reduces the chance of H desorption due to thermal motion and UV irradiation. But the activation barrier for hydrogen atoms to chemisorb on graphitic surfaces is by far too high to occur classically even at temperatures around 200 K. So how is it possible for H atoms to add to interstellar PAHs? Calculations show that the activation barrier for this reaction is clearly reduced at the edge sites of PAHs compared with the binding of H atoms to central PAH carbon atoms [48]. A further help for the addition of H atoms to PAHs can be given by quantum tunnelling of H atoms through the remaining activation barrier. The resulting hydrogen tunnelling rates under the conditions of photon-dominated regions and neutral diffuse clouds have to be included in a full surface reaction network [44,49].

So far we have seen that quantum tunnelling is deeply involved in the astrochemical synthesis of H_2_. The next big step of chemical evolution towards prebiotic chemistry is the formation of water (H_2_O) as one of the most important molecules for life. Is tunnelling also involved in this step? In order to get an answer we have to go back to the coldest regions of the interstellar medium and ask the dark clouds.

Under ultracold conditions within dark clouds (5-20 K) icy mantles are formed on interstellar dust particles. Observations revealed that H_2_O is the most abundant constituent of such icy mantles [50]. As gas-phase synthesis of H_2_O turned out to be too inefficient with respect to the detected abundances it is proposed that H_2_O is formed directly on dust grains via surface reactions [e.g., 51]. Among different surface reactions the following reaction based on already formed H_2_ (see above) is considered to be one of the most common routes [52]:

(12) *OH +H_2_* → *H_2_O+H*

This reaction, however, has a large activation barrier and thus at ultracold temperatures there is a very low probability that OH and H_2_ react via thermal activation. Experiments running under simulated conditions of dark clouds revealed that the efficiency of this reaction is highly influenced by isotope effects. This means that using molecular deuterium (D_2_) instead of H_2 _reduces the efficiency of the discussed reaction by about one order of magnitude [52]. Such observations point to quantum tunnelling due to the strong dependence of the tunnelling rate on the particle mass. Other relevant reaction pathways for the synthesis of solid H_2_O in dark clouds such as the hydrogen peroxide pathway also depend on quantum tunnelling in order to circumvent the low probability of overbarrier reactions [53]. This shows that quantum tunnelling is of high importance for the formation of H_2_O within dark clouds.

But does tunnelling also play a role in the astrochemical formation of larger, more complex molecules of prebiotic relevance? To answer this question, we must consider carbon monoxide and formaldehyde. Carbon monoxide (CO) is the second most abundant molecule condensed on icy dust grains in dark clouds [54], while formaldehyde (H_2_CO) is among the most important prebiotic molecules detected so far in dark clouds. The importance of H_2_CO is mainly due to its role as a precursor for the interstellar formation of amino acids (the building blocks of proteins) and sugars (e.g. ribose, one of the building blocks of DNA) [55-57]. But how is H_2_CO formed?

CO ice grows on top of a water-rich layer on interstellar dust grains when the density increases in a dark cloud [50]. This CO ice can be hydrogenated via the successive addition of hydrogen atoms according to the following sequence [58]:

(13) *CO* → *HCO* → *H_2_CO* → *CH_3_O* → *CH_3_OH*

The sequence shows that the consecutive hydrogenation of CO leads to the formation of H_2_CO. But the first addition reaction of the sequence, the CO → HCO reaction, is faced with a huge activation barrier. As a consequence, the low probability of overbarrier reactions under the ultracold conditions of dark clouds would lead to very low addition reaction rates. Such low reaction rates are not consistent with the observed interstellar abundance of H_2_CO. Experimental and theoretical results revealed that under the ultracold conditions of dark clouds quantum tunnelling becomes the dominant transition mechanism for the H + CO addition, leading to substantial reaction rates of H_2_CO [59,60]. The same case of high activation barrier and the importance of quantum tunnelling is also true for the third addition reaction in this sequence as a precursor to the formation of methanol [59]. From these hydrogenation examples we see that quantum tunnelling also plays an important role for surface reactions towards more complex molecules with high prebiotic relevance.

In summary, it can be stated that quantum tunnelling is a key process at the basis of chemical evolution towards prebiotic chemistry. The tunnelling phenomenon does not only play a crucial role for astrochemistry in stellar interiors but also for cold regions of the interstellar medium. In neutral diffuse clouds and especially in dark clouds quantum tunnelling boosts various surface reactions on interstellar dust grains towards the synthesis of important prebiotic molecules.

## 4 SUB-SURFACE HABITABILITY, GEOTHERMAL ENERGY AND QUANTUM TUNNELLING

We have seen in the previous section that interstellar dust particles of micrometer size are effective quantum tunnelling factories for the astrochemical synthesis of molecules which are relevant for prebiotic chemistry. Interstellar dust particles can aggregate within a few million years to the extend that they build large objects such as asteroids, moons or planets. If we zoom out from the scale of micrometers and look at such celestial bodies with sizes from hundreds to thousands of kilometres, will we still see quantum tunnelling effects relevant for prebiotic chemistry and habitability?

**Fig. (4):**
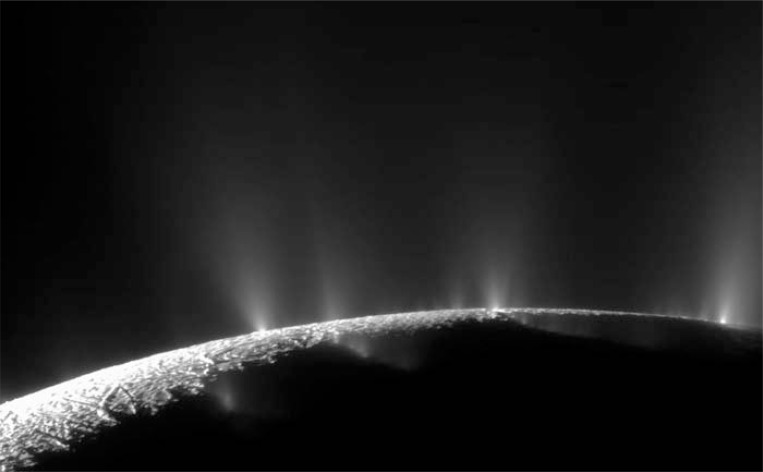
Dramatic plumes, both large and small, spray water ice out from many locations along the famed "tiger stripes" near the south pole of Saturn's moon Enceladus. The tiger stripes are fissures that spray icy particles, water vapour and organic compounds. This mosaic was created from two high-resolution images that were captured by the narrow-angle camera when NASA's Cassini spacecraft flew past Enceladus and through the jets on Nov. 21, 2009. Lit terrain seen here is on the leading hemisphere of Enceladus (504 kilometres, 313 miles across). The view was obtained at a distance of approximately 14,000 kilometres (9,000 miles) from Enceladus and at a Sun-Enceladus- spacecraft, or phase, angle of 145 degrees. Image scale is 81 meters (267 feet) per pixel. Image Credit: NASA/JPL/Space Science Institute.

Enceladus, one of the icy moons of Saturn, is a very extraordinary moon in the solar system. Despite its relatively small mean diameter of 504 km [61] it shows very high endogenic geologic activity. This activity leads to complex surface features and even to plumes of ice particles and water vapour escaping from a system of surface fractures [62] (Fig. **4**). The composition of the ejected particles strongly suggests a liquid water reservoir below the solid surface [63]. Such enormous geologic activity could not be caused by gravitational energy because of the small size of this moon [64]. The activity is even more surprising if we consider tidal heating as a source of energy and compare the conditions for Enceladus with its adjacent moon Mimas. This icy moon is also a few hundreds of kilometres in diameter. With a smaller distance to Saturn and a higher eccentricity of its orbit than Enceladus the effect of heating via tidal deformation should be higher for Mimas. However, there has been no current geologic or thermal activity detected so far on Mimas [65]. So why does Enceladus have such a high activity and probably a liquid water reservoir, making this world potentially habitable? Where does this high amount of internal energy come from?

Recent space probe based investigations and numerical simulations imply that about 60 % of the total mass of Enceladus is rock rather than ice [66]. This rock mass fraction is much higher than that of Mimas whose value is only about 20 % [67] – a fact which becomes important for our question when we look at radioactive isotopes. Such isotopes are concentrated in the rock mass fraction of planetary bodies and release decay energy. The decay energy causes internal radiogenic heating with an intensity directly proportional to the rock mass fraction of icy satellites [68]. Thus, due to the high rock mass fraction of Enceladus, internal radiogenic heating is by far more important for the thermal evolution of this moon than for Mimas. In current models of the thermal evolution of Enceladus, radioactive isotopes with long half-lives play a prominent role. According to these models the decay energy of such isotopes could melt the ice fraction after the formation of Enceladus and lead to planetary differentiation (the creation of a rocky core and a liquid/ice mantle) if favourable conditions were given. But even if the conditions were not so favourable, radiogenic heating of long-lived isotopes could have raised the temperature of the ice fraction up to a level that tidal deformation – and thus tidal heating – became effective to differentiate Enceladus. The long half-lives have kept the internal temperature of this moon above freezing so far by the combination of long-term radiogenic heating and tidal friction [64,67,69,70]. This model explains the difference in geologic activity between Mimas and Enceladus: the much smaller rock fraction of Mimas restricts the value of long-term radiogenic heating to a level which cannot facilitate tidal heating [67]. The described model for the thermal evolution of Enceladus still leaves several questions open [71]. However, the higher rock mass fraction and thus the important role of radiogenic heating by long-lived radioactive isotopes has to be included in all alternative models.

The most important long-lived radioactive isotopes (in terms of their abundance within the rock fraction and amount of released decay energy) are ^235^U, ^238^U and ^232^Th [68]. It is these important heavy isotopes which bring quantum tunnelling back into our discussion. We have seen in section 2 that the fusion of lighter atomic nuclei to form heavier ones is exothermic and releases nuclear binding energy that is equivalent to a mass deficit according to *E=mc^2^.* The released binding energy per nucleon increases with increasing mass numbers, but the steps become smaller with higher mass numbers until a maximum is reached at ^56^Fe [23]. Heavier isotopes cannot be produced via exothermic fusion reactions (such as thermonuclear fusion) because this would consume energy rather than releasing it. But this fact has its flipside: radioactive alpha-decay. This decay releases energy and thus occurs spontaneously. Alpha decay works with heavy isotopes such as uranium and thorium and can be described with the general formula

(14) XZA→YZA−−24+He24

where *X* is the parent isotope, *Y* the daughter isotope, *Z* the atomic number and *A* the mass number [72]. The formula shows that alpha-decay transforms the parent nucleus into a daughter isotope by emitting a ^4^He nucleus (He^2+^). However, when considering at the kinetic energy of emitted He^2+ ^a paradox becomes evident: the potential barrier between the initial state of the parent nucleus and the final state determined by the separated He^2+^ and the daughter nucleus is too high for He^2+^ to overcome the barrier classically [73]. This nuclear force barrier which He^2+^ nucleons feel when trying to separate and escape from a parent nucleus corresponds to the Coulomb barrier for particles which are forced close to a nucleus. The paradox during alpha-decay can thus be resolved similarly to that of the thermonuclear reactions: quantum tunnelling [74,75]. This means that during alpha-decay He^2+^ particles tunnel through the nuclear force barrier out of a heavy nucleus. By comparing the mass of a parent nucleus with the sum of the masses of its daughter nucleus and the separated He^2+^ we see a mass deficit of the decay products. The energy-mass equivalence allows us to calculate the released energy during alpha-decay via

(15) *E* = (*M_X_* – *M_Y_* – *M_He_*) *c*^2^

where *E* is the released energy (referred to as the Q-value in particle physics), *M_x_* the mass of the parent nucleus, *M_y_* the mass of the daughter nucleus, *M_He_* the mass of the emitted He^2+^ and c the velocity of light [72]. It is this high amount of released energy (e.g. 4.25 MeV for the decay of ^238^U) which causes the above mentioned internal radiogenic heating of icy satellites.

Our understanding of decaying uranium and thorium nucleons reveals that quantum tunnelling plays an important role in making Enceladus a geologically active and potentially habitable world: He^2+^ tunnelling causes internal radiogenic heating via the alpha-decay of heavy, long-lived isotopes concentrated in its large rock fraction. This type of heating is high enough that tidal heating becomes effective for Enceladus and contributes to the ongoing high geologic and hydrologic activity which makes this small moon a potentially habitable world.

But is Enceladus one of a kind or could quantum tunnelling turn other solar system bodies into hot spots for our topic?

Let us remain in the Saturnian system for the moment and look at another highly dynamic and enigmatic world – Titan. This moon is outstanding in the solar system due to its similarities with Earth: it has a nitrogen (N_2_) atmosphere like our own planet with a comparable atmospheric surface pressure [76]. Abundant evidence for clouds, episodic rainfall, rivers and even lakes have recently been found by the Cassini/Huygens mission [77,78]. However, this world is by far too cold at its surface (about -180 °C) for water to be available in a liquid state for a hydrologic cycle [76]. In fact, instead of a water cycle we observe a methane cycle on Titan [77]. Methane is a key component in the complex organic chemistry taking place in Titan's upper atmosphere: here, solar ultraviolet radiation and highly energetic particles in Saturn's magnetosphere dissociate methane and initiate the production of larger organic hydrocarbon units such as acetylene, ethane and ethylene [79,80]. These units as well as nitrogenated hydrocarbon species are subsequently coupled into more complex organic compounds which results in the generation of hydrocarbon-nitrile aerosols (tholins) [80, 81] (see also Trainer, 2013, in this issue). Such tholin aerosols slowly sink into the deeper atmospheric layers and finally rain to the surface [82]. Experiments revealed that complex organic aerosols produced under simulated atmospheric conditions of Titan contain nitrogenated aromatic hydrocarbons in the form of Nucleobases and Amino Acids [83]. All these described processes and observations make Titan a hot topic for prebiotic chemistry.

After we have seen how important methane is for initiating the complex organic and possibly prebiotic chemistry in Titan's atmosphere we should search for a reliable source of methane on this moon. The demand is high: methane is constantly consumed by photodissociation and conversion into larger, more complex hydrocarbons as well as by atmospheric escape. The level of consumption is such that the amount of methane in the present day atmosphere would be removed within 30-100 million years [84]. This time frame is short compared to Titan's age of about 4500 million years and raised the question about the source of methane supply capable of maintaining its atmospheric content of several percent until the present day. The observed lakes on Titan's surface are no serious suppliers because their stock of liquid methane is too small [85]. Now, where does the replenishment of atmospheric methane come from? Could it possibly come from Titan's interior? Let's have a closer look at the internal structure of this moon.

Current models about Titan's internal composition based on space probe measurements describe this icy satellite as a differentiated planetary body with a rock mass fraction of about 50-70 % [71]. This high rock mass fraction gives radiogenic heating via the decay of long-lived heavy isotopes a very prominent role in driving the planetary differentiation of this moon and especially in causing and supporting a liquid subsurface water ocean [71,85,86]. It has recently been shown that most of the methane which was initially incorporated in Titan could still be present by having been basically dissolved in the liquid water ocean: although the solubility of methane in water is low, Titan's proposed subsurface ocean is very massive, taking up more than 50 % of the moon's total volume. Therefore the absolute amount of dissolved methane is high. Outgassing of a moderate level of methane from this massive water ocean could thus be sufficient to support the present-day amount of atmospheric methane [85].

When looking at Titan's complex organic chemistry from our quantum point of view we see that tunnelling initiates and supports the chemical evolution in its atmosphere in two different ways: the endogenic support is caused by He^2+^ tunnelling due to the energy release of radioactive alpha-decay. This radiogenic heat has created and mainly supports the proposed subsurface liquid water ocean which stores methane by dissolution such that moderate outgassing can compensate its consumption through prebiotic atmospheric chemistry. The exogenic support can be traced back to nuclear tunnelling which enables thermonuclear reactions in our sun and therefore causes the UV irradation required for the photolysis of atmospheric methane. Thus, both the quantum tunnelling of a particle *out* of a nucleus (the source of internal radiogenic heat) and the tunnelling of a particle *into* a nucleus or forming a nucleus (the source of solar UV irradation) are important factors which turn Titan into a planetary reaction vessel for prebiotic chemistry.

Leaving the Saturnian system, we examine several other planetary bodies which demonstrate He^2+^ tunnelling. Further prominent examples of worlds with possible subsurface water oceans such as the asteroid Ceres [87], the large icy satellites of Jupiter [88-90], Neptune's moon Triton [91] and some large Kuiper belt objects [92] tell us that internal long-lived radionuclide heating via quantum tunnelling is important throughout the solar system.

What about our own planet? Could He^2+^ tunnelling be of any importance for prebiotic chemistry and life on Earth? In the first chapter we have seen that sunlight drives most ecosystems either directly or indirectly via oxygenic photosynthesis or its products. But there is also a dark side of life: hidden deep below the photosphere in places such as the deep sea or the crustal lithosphere some species and ecosystems exist completely independent from sunlight. How do they do that? As life needs free energy to avoid thermodynamic equilibrium and because sunlight as a source of free energy is not available, alternative sources must be tapped.

One strategy pursued by anaerobic green sulphur bacteria is to run anoxygenic photosynthesis using geothermal light [93]. Deep-sea geothermal light is caused basically by thermal radiation from high-temperature hydrothermal vents which transfer geothermal heat from the lithosphere to the surface [94]. Systems of hydrothermal vents can be found at various deep sea locations such as mid-ocean ridges, hotspot volcanoes and seamounts [95]. The hot fluids emanating from such submarine hydrothermal vents create several gradients between the vents and the surrounding sea water ranging from centimetres to metres. Such gradients are described in terms of temperature, redox and pH values and also by chemical gradients caused by large amounts of reduced elements and gases (e.g. Fe^2+^, H_2_, H_2_S, CO_2_ and CH_4_) dissolved in the hot fluids [95-97].

These far-from-equilibrium conditions are very attractive for theories about prebiotic chemistry and the origin of life at hydrothermal vents [97] (see also Lemke, 2013, and Mast *et al.*, 2013, in this issue) and point to another strategy life uses to tap resources of free energy other than sunlight: transferring energy from the geothermal source of hydrothermal vents to higher trophic levels by anaerobic chemolithoautotrophy [95]. With this strategy organic material is produced from CO_2_ and energy is gained by redox reactions between inorganic chemicals supplied by hydrothermal fluids.

Now what is the source of geothermal energy that fuels the described submarine habitats with anaerobic chemolithoautotrophic micro-organisms at the base of the food chain? Recent measurements of the flux of geoneutrinos (electron antineutrinos produced by the decay of radioactive isotopes in Earth's mantle) made with the KamLAND detector of the Kamioka Neutrino Observatory in Japan allow the contribution of radiogenic heat to the total heat flux from Earth's interior to the surface to be estimated. The results indicate that about 50% of Earth's heat flux is currently caused by the decay of ^238^U and ^232^Th [98,99]. This means that a very substantial part of the geothermal energy that generates thermodynamic disequilibrium conditions at hydrothermal vents and thus allows micro-organisms to live completely independently from sunlight can be traced back to He^2+^ tunnelling.

But settling down in deep-sea hydrothermal vent environments is not the only way for life to turn its back on the photosphere: fracture zones within Earth's crust extending to depths of several kilometres below the land surface have been proven to be lithospheric habitats that are completely decoupled from the photosphere and its products [100]. Here, bacterial life has found a way to link anaerobic chemolithoautotrophy directly with the radioactive decay of uranium: the radiolysis of water and bicarbonate molecules induced by uranium oxides generates protons, molecular hydrogen and highly reactive molecular species which interact with various minerals in the surrounding. The products of these interactions are consumed by the bacteria for sulfate reduction, carbon and nitrogen fixation, ATP synthesis and other metabolic processes [101].

In summary, when looking at He^2+^ tunnelling, we see a very important factor for Earth's habitability outside the photosphere. This fact has a vital consequence: the potential subsurface oceans of various planetary bodies all around the solar system – oceans created and/or supported by the impact of He^2+^ tunnelling – become highly interesting in terms of chemical evolution, prebiotic chemistry and exobiology.

## 5 QUANTUM TUNNELLING IN MOLECULAR BIOLOGY

In the previous sections we have seen how quantum tunnelling is important for the topics environment and synthesis. In this section I want to focus on the aspect of “function”. This aspect is involved in the origin of organized complexity of organic molecules such as DNA, proteins and cofactors. Unlike arbitrary complexity (e.g. the chaotic assembly of small crystals to a stone), organized complexity means that a certain molecular structure is formed via specific information and correlates to a specific function within a network (cell, organism). Could quantum tunnelling have a role in these functional molecules?

DNA is a functional polymer with an extraordinary property: the polymerization of nucleotides to DNA is not arbitrary but contains specific information. The information is the result of evolution and codes for protein synthesis as well as other functions required to build an organism. This genetic information is written as specific sequences of nucleic acid base pairs and is transferred via autocatalytic replication to new nucleotide polymers. However, the genetic code in DNA is not completely stable even in the absence of extrinsic damaging factors: a universal spontaneous point mutation bias can be detected with respect to transitions of the base pairs G:C to A:T and A:T to G:C which cannot be sufficiently explained by well-known mutation models such as UV irradiation, oxidative damage or CpG hypermutation [102]. This implies that this kind of point mutation has to be regarded as an inherent characteristic of DNA. How is this possible?

Recent experiments with deuterated bacterial spores revealed that the rates of spontaneous point mutations in cells which are grown in culture media with deuterium oxide (D_2_O) are lower as compared to spontaneous mutations rates in water (H_2_O) based culture media [103]. This means that we see a dependence of the mutation bias on the involved particle mass. In section 1 we have seen that quantum tunnelling rates strongly depend on the particle mass. We have also seen in section 3 that this dependence is used to check if quantum tunnelling is important for certain chemical reactions (isotope effect). Could the detection of an isotope effect on spontaneous mutations rates tell us that quantum tunnelling plays a significant role in the universal point mutation bias in DNA?

The Löwdin DNA mutation model [104] pays attention to the quantum nature of protons involved in the hydrogen bonds between complementary base pairs. Löwdin's model is based on the fact that stable Watson-Crick base paring and thus stable genetic information requires the precondition that protons don't change their position in the base pairs. The position of protons in a base pair is defined by the canonical form of the involved nucleic base molecules. However, the Löwdin model states that due to quantum tunnelling through proton transfer barriers, the position of protons within a nucleic base can change with a small probability when the protons are in a quantum mechanically nonstationary state immediately after a DNA replication. As a consequence of quantum tunnelling, the canonical form of a nucleic base is transformed into its tautomeric form which doesn't bind to the initial base pair partner. But the tautomeric form can bind to a different base. This process and subsequent steps ultimately leads to point mutations in DNA.

The Lödwin model describes the universal point mutation bias as a result of quantum tunnelling of protons in DNA. This means that proton tunnelling influences the main function of DNA – the reliable storage of genetic information. Publications referring to the Löwdin mutation model have become numerous in recent times. A most recent collection of theoretical studies is given by Cooper [105], and a collection of genomic analysis reports supporting the Löwdin model has been made by Lu [103].

Ironically quantum tunnelling is not only discussed as an intrinsic damaging factor to DNA but is also involved in DNA repair [106]. The key factor which enables DNA repair is electron tunnelling. How does such a repair mechanism work? Ultraviolet irradiation can damage DNA by inducing the formation of bulges in DNA strands. These bulges are caused by the dimerisation of adjacent pyrimidines on a DNA strand in reaction to UV irradiation. The flavoprotein photolyase is able to repair such deformed DNA by splitting the covalentely linked pyrimidines via electron transfer so they can return to their normal monomeric form and no longer form a dimer. To do this repair job, photolyase needs visible light. 

It sounds strange that light is used to repair a light induced damage, but the situation becomes clear if we look at the wavelengths: one of the cofactors of photolyase is an antenna complex which absorbs light with longer wavelength (blue) than the UV radiation. The absorbed light is important as it leads to an excitation of the antenna complex. The excitation is then transferred to the other cofactor of photolyase, the redox cofactor FADH (Flavin Adenine Dinucleotide). The function of FADH^-^ is to transfer the electron to the antibonding molecular orbital of the DNA pyrimidine dimer. After the transferred electron has done its repair job to split the dimer it returns to the redox cofactor. But how is the electron transferred from FADH^-^ to the pyrimidine dimer? A closer look at the coupling between photolyase and DNA reveals an unexpected situation: FADH^- ^couples most strongly to the dimer with its adenine part. The coupling parameter influences the redox reaction rate which shows that adenine is indeed involved in the electron transfer process. But, however, it is the flavin part of the FADH^-^ where the electron is located prior to its transfer. When considering the intramolecular redox conditions of FADH^-^ and experimental results on the structural behaviour of FADH^-^, the electron transfer cannot be explained by a jump of the electron from the flavin part to the adenine part and then to the dimer (multistep hopping mechanism). 

So, how does the redox reaction take place in such a situation? The answer is superexchange-mediated tunnelling over long distances [106-108]. This process makes it possible that quantum tunnelling of electrons in proteins can occur over distances of up to three nanometres [109,110] although such a potential barrier width is much larger than possible widths in vacuum (see section 1). But low-lying electronic states provided by the protein via a superexchange-mediated mechanism boost electron transfer rates so they are still relatively high over such large distances [108]. Applied to our example, this means that an electron is directly transferred from the flavin part to the dimer via single-step long-range quantum tunnelling. The repair of UV-damaged DNA by the protein photolyase is thus essentially based on long-range electron tunnelling.

The above mentioned quantum tunnelling processes in molecular biology have been chosen as examples for a detailed discussion as they clearly demonstrate that both nuclear tunnelling and electron tunnelling can influence and enable biomolecular functions and that functions of both DNA and proteins/cofactors are involved.

But there are more such cases of quantum tunnelling in biology. Long-range electron tunnelling is also crucial for many other types of proteins with functions based on redox-reactions [111]. Important examples are cellular respiration [112] and photosynthesis [113]. In enzyme-catalysed reactions hydrogen tunnelling is widely discussed [114]. Enzymes are primarily proteins with specific catalytic functions. This means that they enhance the rate of specific reactions in biomolecular syntheses relative to reaction rates in their absence [115,116]. How are they doing this is still not well understood and a comprehensive model of catalysis is one of the big challenges in chemical biology [117,118]. The lack of a comprehensive model causes intense debates about e.g. the role of protein dynamics [115,116] and the role of hydrogen tunnelling in enzyme-catalysed reactions [118-120]. Now, what can be stated so far about hydrogen tunnelling in enzyme-catalysed reactions? Large isotope effects are observed in many enzymatic reactions that involve hydrogen transfer [117,121,122]. These observations indicate that tunnelling occurs in enzymatic reactions. But what is its role? A most recent and thorough review [118] concludes that in enzyme-catalysed reactions tunnelling is probably only weakly catalytic. This means that it is not significantly *enhancing* reaction rates. But hydrogen tunnelling is an important *component* of reaction rates so the reactions would otherwise be much slower.

In summary tunnelling has guided us via prominent examples to biochemistry where quantum tunnels are important infrastructures in the world of functional molecules: spontaneous mutations in DNA, enzymatic reactions and especially energy transduction pathways are highly influenced, supported and even enabled by quantum tunnelling. The transition probability of the tunnelling concept from the field of organic chemistry [123] to biochemistry [124] has significantly increased within the past years while, at the same time, the conceptual barrier between chemistry and biology has become smaller and has begun to disappear [125] (see also Pross, 2013, in this issue).

## CONCLUSION

This review was a round trip from hot stellar interiors to cold interstellar medium, from deep lithospheres and subsurface oceans to planetary upper atmospheres and from the microcosm of biomolecular nanomachines to the evolution of macroscopic multicellular life. At each stop we have seen that quantum tunnelling is of vital importance for life and its origin. These stops are not isolated but rather closely connected with each other. The following example further clarifies this concept by focusing on photosynthesis as an example.

Photosynthesis is basically performed by functional biomolecules whose redox reactions rely on long-range electron tunnelling. All elements which build these biomolecules (except hydrogen) were produced by thermonuclear reactions in stars via nuclear tunnelling. Photosynthesis in deep sea environments uses geothermal light where half of its energy can be traced back to the effect of He^2+^ tunnelling during radioactive alpha decay of heavy elements within the lithosphere. In contrast, the energy source of oxygenic photosynthesis is sunlight which is a product of nuclear tunnelling in thermonuclear reactions. Our sun itself was formed by the collapse of dark interstellar clouds. In this process, molecular hydrogen plays an important role and is formed in dark clouds by the help of quantum tunnelling. Oxygen, the important byproduct of oxygenic photosynthesis, is used by multicellular lifeforms for cellular respiration – a process which relies on long-range electron tunnelling. The evolution of all multicellular lifeforms is based on DNA mutations and energy input over billions of years. A source of mutations is proton tunnelling in DNA while the large period of time of solar energy input is due to specific characteristics of quantum tunnelling taking place in thermonuclear reactions.

Although this example does not cover every single topic previously discussed, it illustrates the main message of this review: there is a highly multidisciplinary network of quantum tunnels essential for the origin and evolution of life. Tunnelling is essential because it

- *provides* different sources of constant *energy* flux over a long period of *time* and thus makes highly advanced complexification in molecular and biological evolution possible,
- *opens* *synthesis* pathways for prebiotic astrochemical reactions which would otherwise not occur,
- *enables or influences* specific *functions* of biomolecular nanomachines maintaining the process of life.
